# Stability of gross primary productivity and its sensitivity to climate variability in China

**DOI:** 10.3389/fpls.2024.1440993

**Published:** 2024-09-06

**Authors:** Xiaojuan Xu, Fusheng Jiao, Jing Liu, Jie Ma, Dayi Lin, Haibo Gong, Yue Yang, Naifeng Lin, Qian Wu, Yingying Zhu, Jie Qiu, Kun Zhang, Changxin Zou

**Affiliations:** ^1^ Ecological Protection and Restoration Center, Nanjing Institute of Environmental Sciences, MEE, Nanjing, China; ^2^ School of Geography, Nanjing Normal University, Nanjing, China; ^3^ College of Urban, and Environmental Sciences, Peking University, Beijing, China

**Keywords:** gross primary productivity, sensitivity, stability, climate change, ecological engineering

## Abstract

Identifying the stability and sensitivity of land ecosystems to climate change is vital for exploring nature-based solutions. However, the underlying mechanisms governing ecosystem stability and sensitivity, especially in regions with overlapping ecological projects, remain unclear. based on Mann-Kendall, stability analysis method, and multiple regression method, this study quantified the stability and sensitivity of gross primary productivity (GPP) to climate variables [temperature, vapor pressure deficit (VPD), soil moisture, and radiation] in China from 1982 to 2019. Our findings revealed the following: (1) GPP demonstrated an increased trend with lower stability in Eastern regions, whereas a decreasing trend with higher stability was observed in Western and Southwest China. Notably, the stability of GPP was highest (74.58%) in areas with five overlapping ecological projects: Grain to Green, Natural Forest Resource Protection Project, Three-River Ecological Conservation and Restoration Project, Return Grazing to Grassland Project, and Three-North Shelter Forestation Project. (2) In regions with minimal or no overlapping ecological projects, temperature and radiation jointly dominated GPP variations. In contrast, water-related factors (VPD and soil moisture) significantly affected GPP in areas with multiple overlapping ecological projects. (3) In the southwestern and northeastern regions, GPP exhibited the highest sensitivity to climate change, whereas, in the eastern coastal areas and Tibet, GPP showed low sensitivity to climate change. In the Loess Plateau, where five ecological projects overlap extensively, carbon sinks primarily demonstrate a monotonic increasing trend, high stability, and low sensitivity to climate change. This study aimed to assess the stability of the land ecosystems and delineate their sensitivity to climate changes, thereby laying the groundwork for understanding ecosystem resilience.

## 1 Introduction

Understanding the stability and sensitivity of terrestrial ecosystems is crucial for accurate predictions of ecosystem dynamics and for informing policies to mitigate climate change (Seddon et al., 2016; Pennekamp et al., 2018; Wang et al., 2022). Ecosystem stability plays a key role in regulating the terrestrial carbon cycle and atmospheric carbon dioxide levels (Messori et al., 2019). With the increasing frequency of extreme weather events due to climate change, inter-annual fluctuations in vegetation growth are rising, indicating a decline in ecosystem stability (Holden et al., 2014; Hu et al., 2023; Zhang et al., 2024b). Sensitivity is defined as the extent to which an ecosystem responds to disturbance and the duration, and it remains in its original state varies across ecosystems (Hu et al., 2021; Li et al., 2022). Differences in ecosystem sensitivity to climate change can disrupt ecological interactions, threatening ecosystem functioning (Walker et al., 2004; Carlson et al., 2012; Thackeray et al., 2016; Nikinmaa et al., 2020; Chen et al., 2022). Studying both stability and sensitivity together provides a comprehensive understanding of ecosystem dynamics, offering robust scientific support for conservation strategies and climate change mitigation efforts (Piao et al., 2020). Ecosystems with lower stability exhibit heightened responses to disturbance and greater sensitivity to environmental perturbations (Hu et al., 2021). Therefore, identifying regions of ecosystem instability and high ecological sensitivity is essential for pinpointing areas vulnerable to ecological change (Thackeray et al., 2016).

Ecosystem gross primary productivity (GPP), which represents the cumulative photosynthetic carbon sequestration by all leaves measured at the ecosystem scale, denotes the comprehensive uptake of CO_2_ within the ecosystem (Anav et al., 2015; Cheng et al., 2017). GPP is a vital indicator of the terrestrial carbon cycle, marking the beginning of carbon sequestration in ecosystems and providing a robust measure of terrestrial carbon uptake (Anav et al., 2015; Xu et al., 2022). The stability and sensitivity of GPP introduce significant uncertainty regarding the resilience and resistance of ecosystems (Liu et al., 2023a). Under minimal or undisturbed conditions, GPP exhibited high stability with negligible fluctuations (Lin et al., 2023). Conversely, when external disturbances exceed GPP’s threshold tolerance, it becomes destabilized and more sensitive to the external environment, making it highly susceptible to climate change (Chen et al., 2017). Therefore, it is imperative to understand the stability of GPP and its sensitivity to environmental variables.

Studies have demonstrated the significant role of climate variables as key regulators of ecosystem processes, making them pivotal external drivers (Qiao and Xia, 2024; Wang et al., 2024). Consequently, the stability and sensitivity of GPP to climate change are essential components of terrestrial ecosystem dynamics (Anav et al., 2015; Chen et al., 2017; Gao et al., 2018; Piao et al., 2019; Xu et al., 2019). Prior research has identified temperature, radiation, soil moisture, and vapor pressure deficit (VPD) as the primary drivers of GPP variations (Nord-Larsen et al., 2019; Anderegg et al., 2020; Hu et al., 2023; Zhang et al., 2024a). Elevated temperatures significantly stimulated GPP until reaching an optimal threshold (He et al., 2022b; Kan et al., 2023). Increased solar radiation was the principal contributor to the observed rise in GPP (Cui et al., 2021). Warming enhances leaf photosynthesis under high soil moisture conditions, whereas drought impedes it (Dong et al., 2022; Guo et al., 2023). In regions with limited water availability, air drying inhibits GPP increase, leading to reduced vegetation growth. Furthermore, heat-induced elevation in VPD has been linked to accelerated vegetation mortality at the forest-grassland interface (Konings et al., 2017; He et al., 2022a). In semi-arid and arid regions, vegetation responsiveness to soil moisture has significantly increased over time (Shen et al., 2021; Li et al., 2022). However, prior research has primarily focused on elucidating the driving mechanisms behind GPP trends, with limited clarity on GPP sensitivity to climate variables (Hooper et al., 2017; Li et al., 2018).

China has undertaken several major projects to safeguard and rehabilitate ecosystems, including the Three North Shelterbelt Forestation Project, the Grain to Green Project, the Natural Forest Resource Protection Project, the Beijing-Tianjin Sandstorms Source Control Project, the Yangtze River Shelterbelt Forestation Project, the Pearl River Shelterbelt Forestation Project, the Return Grazing to Grassland Project, the Three-River Ecological Conservation and Restoration Project, and the Desertification Control in the Karst region of Southwest China (Niu et al., 2022; Chang et al., 2023). Currently, 17.42% of the vegetated land is affected by one ecological project, and 78.87% of the area is affected by the overlapping effects of multiple ecological projects (Shao et al., 2023). Existing studies primarily focused on the carbon cycle in a single project, neglecting the overlapping effects (Niu et al., 2023; Shao et al., 2023). There is an urgent need to elucidate the stability, driving mechanisms, and sensitivity of GPP in both singular and overlapping ecological project areas (Shao et al., 2023). This endeavor is crucial not merely for evaluating carbon sequestration potential and sink enhancement within major ecological ventures but also for achieving the “carbon neutrality” goal.

This study aimed to enhance our understanding of the sensitivity of GPP to climate variables in overlapping ecological engineering regions, focusing on two primary aspects: the stability of GPP and its sensitivity to climate change. To achieve these, we first analyzed the trend change and stability change of GPP from 1982 to 2019 in overlapping ecological engineering areas. Second, we explored the driving mechanisms of the GPP in overlapping ecological engineering areas. Finally, we revealed the sensitivity of GPP to climate change in overlapping ecological engineering areas. This research enhances comprehension of ecosystem sensitivity to climate change and contributes to an improved understanding of ecosystem stability.

## 2 Materials and methods

### 2.1 Study area

**Figure 1:**
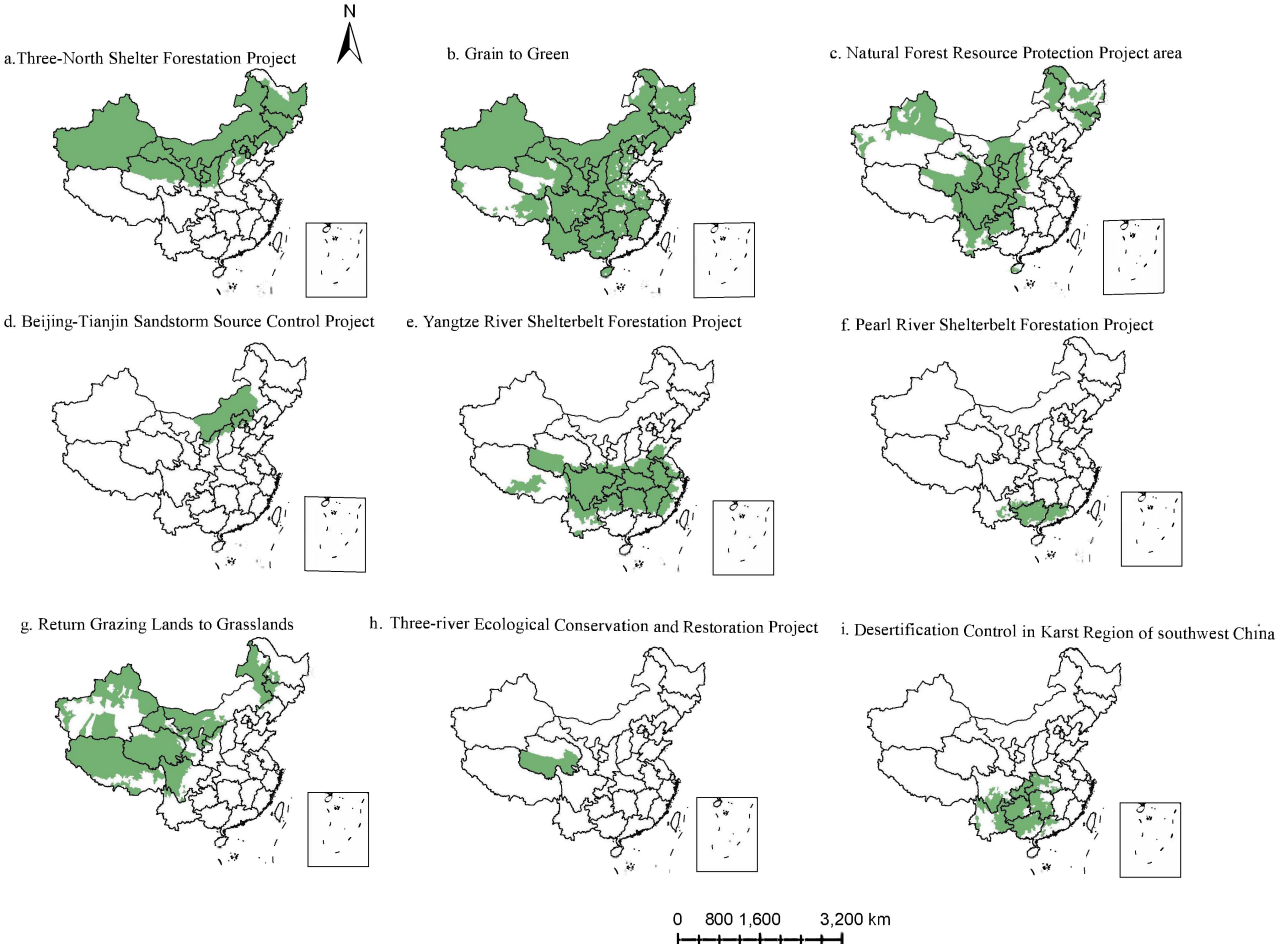
The spatial distribution of the nine ecological engineering. **(A)** Three-North Shelter Forestation Project; **(B)** Grain to Green; **(C)** Natural Forest Resource Project area; **(D)** Beijing-Tianjin Sandstorm Source Control Project; **(E)** Yangtze-River Shelterbelt Forestation Project; **(F)** Pearl River Shelterbelt Project. **(G)** Return Grazing Land to Grasslands; **(H)** Three-river Ecological Conservation and Restoration Project; **(I)** Desertification Control in Karst Region of southwest China.

Since the late 1970s, China implemented a series of ecological projects, including the Three North Shelterbelt Forestation Project, the Grain to Green Project, the Natural Forest Resource Protection Project, the Beijing-Tianjin Sandstorms Source Control Project, the Yangtze River Shelterbelt Forestation Project, the Pearl River Shelterbelt Forestation Project, Return Grazing to Grassland Project, the Three-River Ecological Conversation and Restoration Project, and the Desertification Control in Karst region of Southwest China (**Figure 1**), with a total area of ecological engineering zones of about 9.3 × 10^6^ km^2^ (Shao et al., 2023). The boundaries of the nine engineering zones were downloaded from the National Ecological Science Data Center (http://www.nesdc.org.cn/).

**Figure 2:**
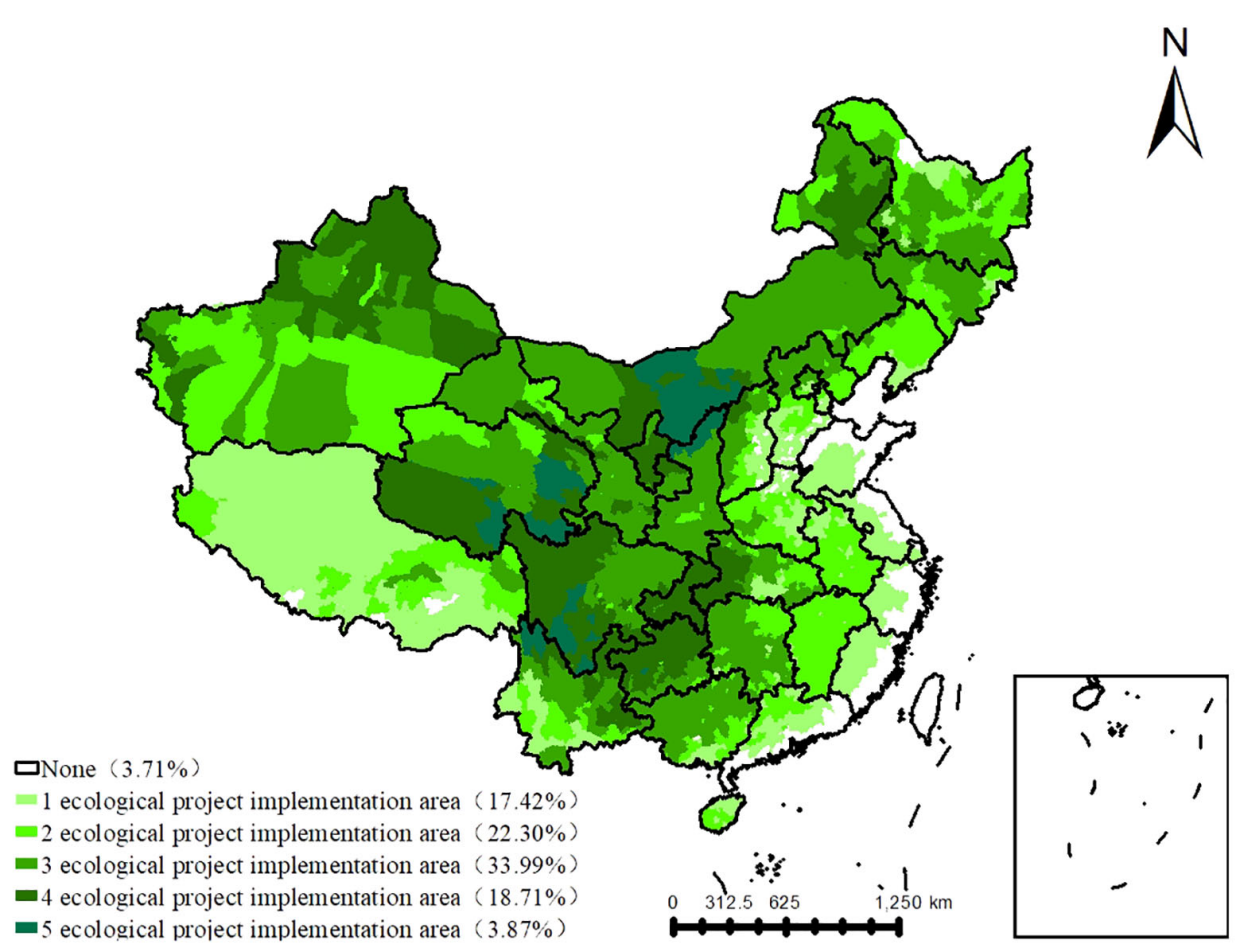
The spatial distribution of the overlapped ecological engineering.

Based on the spatial convergence of nine major ecological project implementation scopes (**Figure 2**), nearly 96.29% of China’s land area lies within ecological project implementation zones, which may be a single ecological works implementation area or multiple ecological works overlapping implementation areas. Conversely, only 3.71% of the territory lacks ecological project implementation, primarily concentrated along the southern coast. One ecological engineering project implementation zones cover approximately 17.42% of China’s territory, predominantly distributed along the eastern coast and encompassing the Return Grazing Lands to Grasslands initiative in Tibet. Overlapping implementation areas of ecological engineering projects span approximately 78.87% of the national land area. Notably, the largest overlapping areas involve three ecological projects, encompassing approximately 33.99% of China’s land area, predominantly situated in central regions such as the Grain to Green Project, the Yangtze River Shelterbelt Project, and the Desertification Control in Karst Regions of Southwest China. Additionally, the most extensive overlaps involve five ecological projects, constituting around 3.87% of China’s land area, concentrated in the Three River Region and Yellow River Basin.

### 2.2 Data sources

We collected the GPP from the Breathing Earth System Simulator version 2.0 (BESS v2.0) in the period of 1982–2019 at a spatial resolution of 0.05° (https://www.environment.snu.ac.kr/bessv2). BESS is a coupled remote-sensed and process-based model. This model was developed to qualify global land-atmosphere flux exchange by integrating key physical and biochemical processes (Jiang and Ryu, 2016; Li et al., 2023). This GPP product was consistent with flux observations and showed a better performance than other remote-sensed GPP products (Chang et al., 2024). First, a two-leaf, two-stream canopy radiative transfer model was used to calculate absorbed photosynthetically active radiation and near-infrared radiation in sunlit and shaded canopies. Then, the relative proportions of C3 and C4 plants were determined by a plant functional type–dependent look-up table method. An optimality-based model was used to quantify the maximum canopy carboxylation rate of C3 and C4 plants at a standardized temperature (25°C). Next, a revised Farquhar model was adopted to compute GPP for sunlit and shaded C3 and C4 plant canopies separately in an iterative manner. Finally, the sum of the relative GPP fixed by C3 and C4 plants was computed to determine the carbon flux quantity in each land grid cell.

The climatic datasets utilized in this study, named TerraClimate, comprised essential variables including air temperature (TEM), soil moisture (SM), VPD, and downward shortwave radiation (RAD). These datasets were procured from the Climatology Lab (Abatzoglou et al., 2018). Acquired from three distinct sources—WorldClim, Climate Research Unit (CRU), and Japanese 55-year Reanalysis (JRA-55), the climatic datasets underwent refinement using the Multivariate Adaptive Constructed Analogs technique to enhance spatial resolution through downscaled monthly time series. This approach has demonstrated superiority over direct daily interpolated bias correction methods, particularly in regions characterized by intricate topography (Jiao et al., 2024). Offering monthly climate insights and climatic water balance dynamics for terrestrial surfaces worldwide, the climatic datasets span from 1958 to 2020. Notably, the data are accessible at a spatial resolution of 1/24°, approximately equivalent to 4 km.

### 2.3 Methods

The Mann–Kendall test is a non-parametric statistical method used to detect monotonic trends in time series data. This method does not require the data to adhere to a specific distribution nor does it assume a linear relationship (Mann, 1945). It has significant advantages in identifying trends in time series data, particularly when dealing with complex, nonlinear data (Liu et al., 2016). Consequently, this study employs the Mann–Kendall method to analyze trends in GPP.

For the GPP time series, the statistic $S_{k}$ is defined as follows (Chen et al., 2012):


(1) $$S_{k}=\sum_{i=1}^{k}r_{i},(k=2,3,\cdots ,n)$$

with


(2) $$r_{i}=\{\begin{array}{c} 1,x_{j}-x_{i}>0\\ 0,x_{j}-x_{i}\leq 0 \end{array},(j=1,2,\cdots ,i)$$

In Equation 2 $x_{i}$ and $x_{j}$ are the *i*th data value in time series.

The GPP trends can be identified by utilizing the standard normal test statistic ( $UF_{k}$ ), which can be calculated as follows:


(3) $$UF_{k}=\frac{\left[S_{k}-E(S_{k})\right]}{{\left[Var(S_{k})\right]}^{1/2}},(k=2,3,4,\cdots n)$$

where UF_1_ = 0. $E(S_{k})$ and $Var(S_{k})$ are the mean and variance of $S_{k}$ , which can be calculated as follows:


(4) $$E(S_{k})=n(n-1)/4$$

(5) $$Var(S_{k})=n(n-1)(2n+5)/72$$

Positive (negative) values of $UF_{k}$ indicate GPP exhibits increasing (decreasing) trends. A typical significance level of α=0.05 was used in the test with $UF_{1-\alpha /2}$ =1.96. If |*UF_k_* |>1.96, then GPP passed the significant test. The Mann–Kendall test was done in MATLAB R2019.

The stability of GPP was evaluated by calculating the coefficient of variation (Zhang et al., 2019). All factors were de-trended before stability analysis (Lin et al., 2023).


(6) $$GPP_{detrend,year}\;=\;GPP_{year}\;-GPP_{trend,year}\;+\;GPP_{mean\;}$$

(7) $$GPP_{trend,year}\;=\;a\times year+b$$

where ${\text{ GPP}}_{year}$ is the raw annual GPP data; $GPP_{trend,year}$ is the GPP trend with each year, and its coefficients are calculated by the linear method. We denote the size of the variable by ${\text{GPP}}_{mean\;}$ , which is averaged from ${\text{ GPP}}_{year}$ . The de-trended variable for each year is denoted by $GPP_{detrend,year}$ . The stability of GPP was then calculated from the de-trended data:


(8) $$GPP_{stability}=\frac{\sqrt{\frac{\sum {\left(GPP_{detrend,year}-GPP_{mean\;}\right)}^{2}}{N}}}{GPP_{mean\;}}$$

The larger $GPP_{stability}$ , the more volatile GPP and less stable it is. In addition, based on natural interval methods, we also classified the GPP stability into five levels (stability, relatively stable, moderate stability, relatively unstable, and unstable). The natural interval method relies on inherent data groups.

To assess the impacts of four climate variables (TEM, VPD, SM, and RAD) on the GPP variations, we employed a multiple linear regression model to compute the linear associations between de-trended GPP and de-trended climatic time series (Equation 9):


(9) $$GPP=a\times S_{TEM}+b\times S_{VPD}+c\times S_{SM}+d\times S_{RAD}$$

where GPP indicated the standardized anomalies of GPP; *S_TEM_*, *S_VPD_*, *S_SM_*, and *S_RAD_* represented the standardized anomalies of temperature, VPD, soil moisture, and radiation; *a*, *b*, *c*, and *d* were the corresponding standardized regression coefficients of the four climatic factors

The magnitude of each standardized regression coefficient indicated the relative significance of the driving forces. In this study, the largest regression coefficients were the main driving factor on the GPP. The climate weights for each driver were reshaped to a range of 0 to 1 (using the minimum and maximum value of any of the climate coefficient values) to further calculate the ecological sensitivity index.

**Figure 3:**
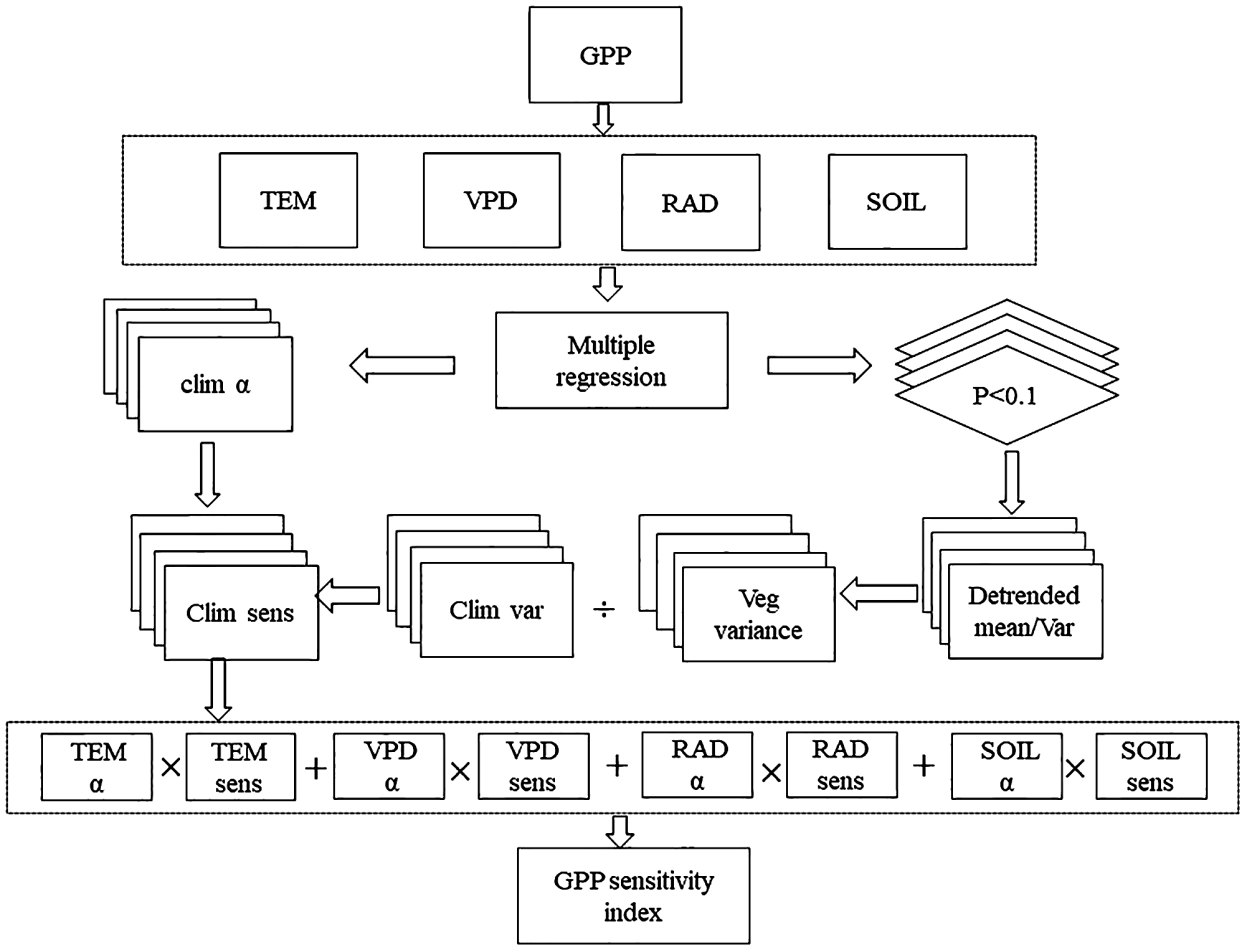
The flowchart for calculating the stability and sensitivity index of GPP.

We constructed a flowchart (**Figure 3**) to illustrate the stability and sensitivity analysis. The sensitivity index of GPP in this study was a proxy of the sum of the response magnitude of vegetation to climate changes. To evaluate the sensitivity index of GPP, all variables were de-trended by subtracting the trend from the original data. The variance of GPP and its driving factors was then computed from 1982 to 2019. The residuals obtained from the linear approach were utilized to establish the mean-variance connection for both GPP and climate variables at each pixel. The residuals were normalized to a scale of 0 to 100 for each variable. Sensitivity metrics were calculated as the logarithm base log_10_-transformed ratios of GPP variability and each of the climate variables. Each ratio was then multiplied by the climate weight, indicating the significance of the climate variable in influencing the variability of the GPP (Seddon et al., 2016). Finally, the sensitivity index of GPP was qualified by the sum of the weighted ratio of air temperature, soil moisture, VPD, and radiation (**Figure 3**). Data analyses were conducted using MATLAB R2019b and ArcGIS 10.7.

## 3 Results

### 3.1 The trend and stability of GPP

**Figure 4:**
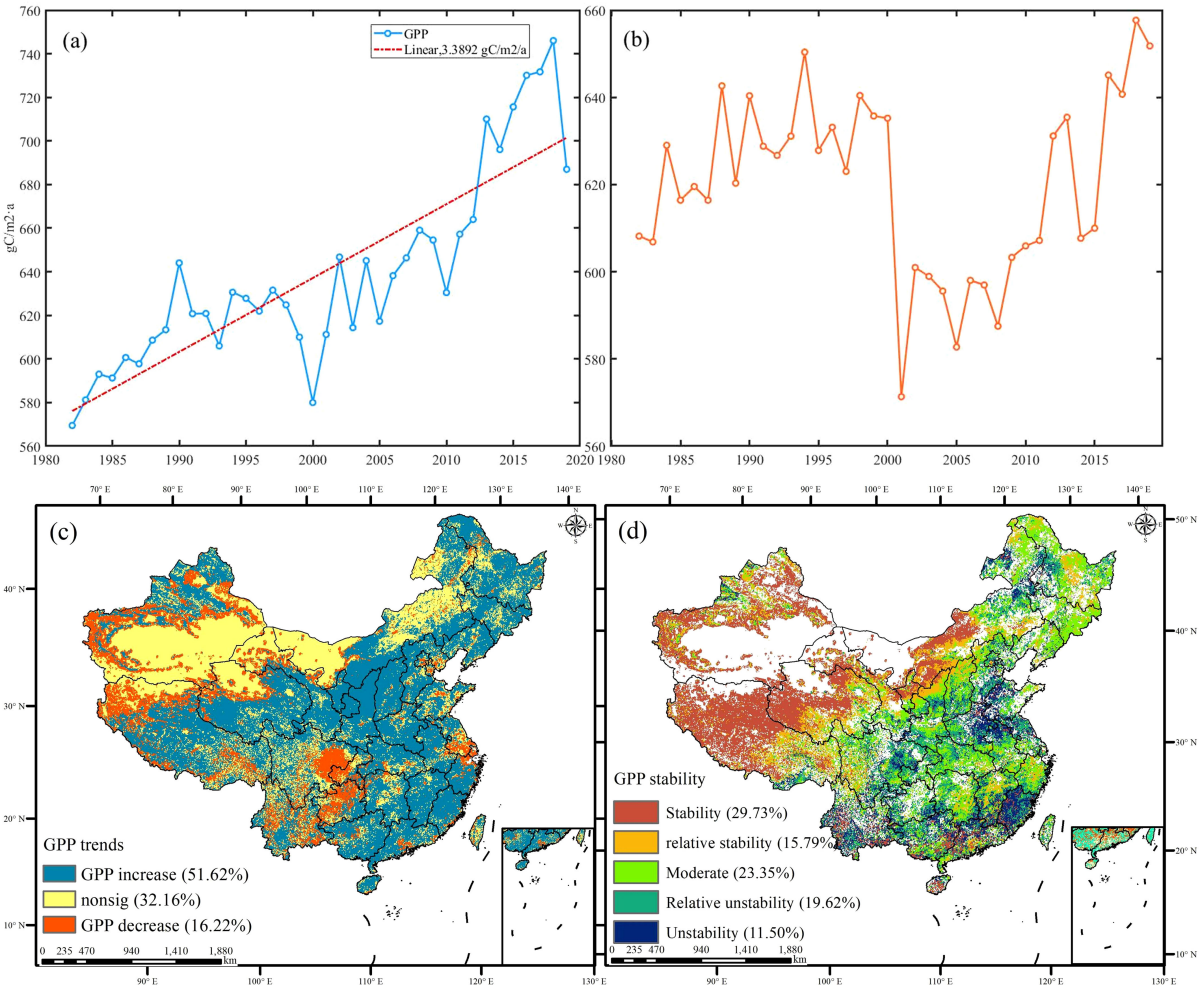
The trend and stability of GPP from 1982 to 2019 in China [**(A)** the linear trend of GPP, **(B)** the inter-annual fluctuation of GPP, **(C)** the spatial distribution of GPP, and **(D)** the stability of GPP].

As shown in **Figure 4A**, GPP had a significant trend from 1982 to 2019, with an increased rate of 3.3892 gC m^−2^ year^−1^. Before 2000, GPP exhibited relatively smooth inter-annual fluctuations; however, after 2000, GPP showed significant increasing trends with sharp inter-annual fluctuations (**Figure 4B**). A total of 65.74% of the GPP in China experienced increasing trends from 1982 to 2019, especially in the pastoral ecological zones in the northeast of China, the Loess Plateau, the Qinling, and the southeast coastal areas (**Figure 4C**). The region was consistent with the key ecological restoration projects, such as the Grain to Green projects and the Natural Forest Protection project. However, in the arid and semi-arid regions of the northwest, the southwest, and the low reaches of the Yangtze River, the GPP had decreased trends. **Figure 4D** highlighted that the instability of GPP was primarily concentrated in the North China Plain, the Southeast Coast, and the Southwest Karst region, whereas GPP demonstrated greater stability in the Qinghai-Tibetan Plateau and arid and semi-arid regions.

**Figure 5:**
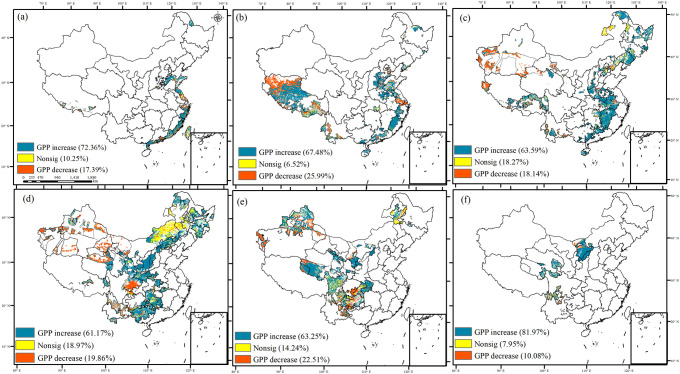
The trend of GPP from 1982 to 2019 in ecological engineering areas [**(A)** zero ecological project areas, **(B)** one ecological project area, **(C)** two ecological project areas, **(D)** three ecological project areas, **(E)** four ecological project areas, and **(F)** five ecological project areas].

In the ecological engineering areas (**Figure 5** and **Table 1**), GPP with greatest percentage of increasing trends in the region with the highest number of overlapping ecological projects, reaching 81.97%. These areas were mainly located in the overlapping implementation of five ecological projects, such as the Grain to Green, the Natural Forest Resource Protection Project, the Three-River Ecological Conservation and Restoration Project, and the Three-North Shelter Forestation Project. The percentage of increasing trends in GPP was relatively higher in the Southeast Coastal Region, which showed a 72.36% increase in GPP. The favorable hydrothermal conditions along the eastern coast were conducive to vegetation carbon sequestration and sink enhancement. In addition, relatively high percentage increases in GPP were observed in regions with the implementation of a single ecological project, particularly in the eastern part of the Grain to Green area and the western part of the Return Grazing to Grassland area. Conversely, areas where two or three ecological programs overlapped experienced lower rates of increase in GPP. This can be attributed to the relatively poor hydrothermal conditions in the northwestern region, resulting in GPP dominated by insignificant changes and exhibiting a lower rate of increase compared to the eastern region.

**Table 1:** The trend of GPP from 1981 to 2019 in ecological project areas.

Overlapping ecological projects	Increasing trends	Non-sig	Decreasing trends
0 ecological project	72.36%	10.25%	17.39%
1 ecological project	67.48%	6.52%	25.99%
2 ecological projects	63.59%	18.27%	18.14%
3 ecological projects	61.17%	18.97%	19.86%
4 ecological projects	63.25%	14.24%	22.51%
5 ecological projects	81.97%	7.95%	10.08%

**Figure 6:**
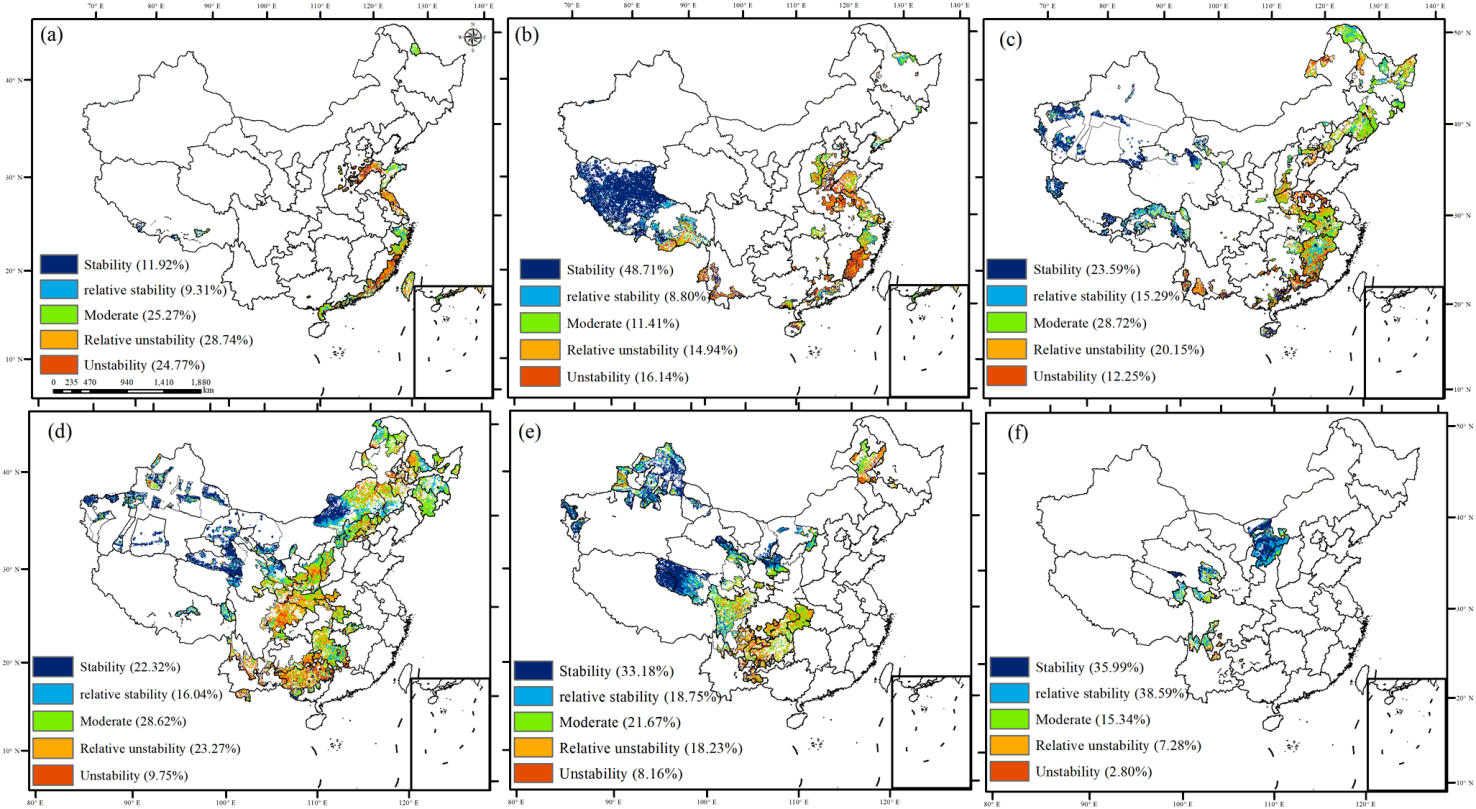
The stability of GPP from 1981 to 2019 in ecological project areas [**(A)** zero ecological project areas, **(B)** one ecological project area, **(C)** two ecological project areas, **(D)** three ecological project areas, **(E)** four ecological project areas, and **(F)** five ecological project areas].

In the ecological engineering areas, the highest percentage of stability in GPP was in the implementation areas of five overlapping ecological engineering regions, accounting for 74.58% (**Figure 6**; **Table 2**). These areas were mainly located in the areas of overlapping ecological projects such as Grain to Green, Natural Forest Resource Protection Project, Three-River Ecological Conservation and Restoration Project, and Three-North Shelter Forestation Project. The second high percentage of stability in GPP was mainly concentrated in the Tibetan with Return Grazing Lands to Grasslands. However, in no ecological project, as well as two or three overlapping ecological projects, the percentage of instability in GPP was relatively low.

**Table 2:** The stability of GPP from 1981 to 2019 in ecological project areas.

Overlapping ecological projects	Stability	Relative stability	Moderate	Relative instability	Instability
0 ecological project	11.92%	9.31%	25.27%	28.74%	24.77%
1 ecological project	48.71%	8.80%	11.41%	14.94%	16.14%
2 ecological projects	23.59%	15.29%	28.72%	20.15%	12.25%
3 ecological projects	22.32%	16.04%	28.62%	23.27%	9.75%
4 ecological projects	33.18%	18.75%	21.67%	18.23%	8.16%
5 ecological projects	35.99%	38.59%	15.34%	7.28%	2.80%

### 3.2 The dominant role of four climate variables in GPP variations

**Figure 7:**
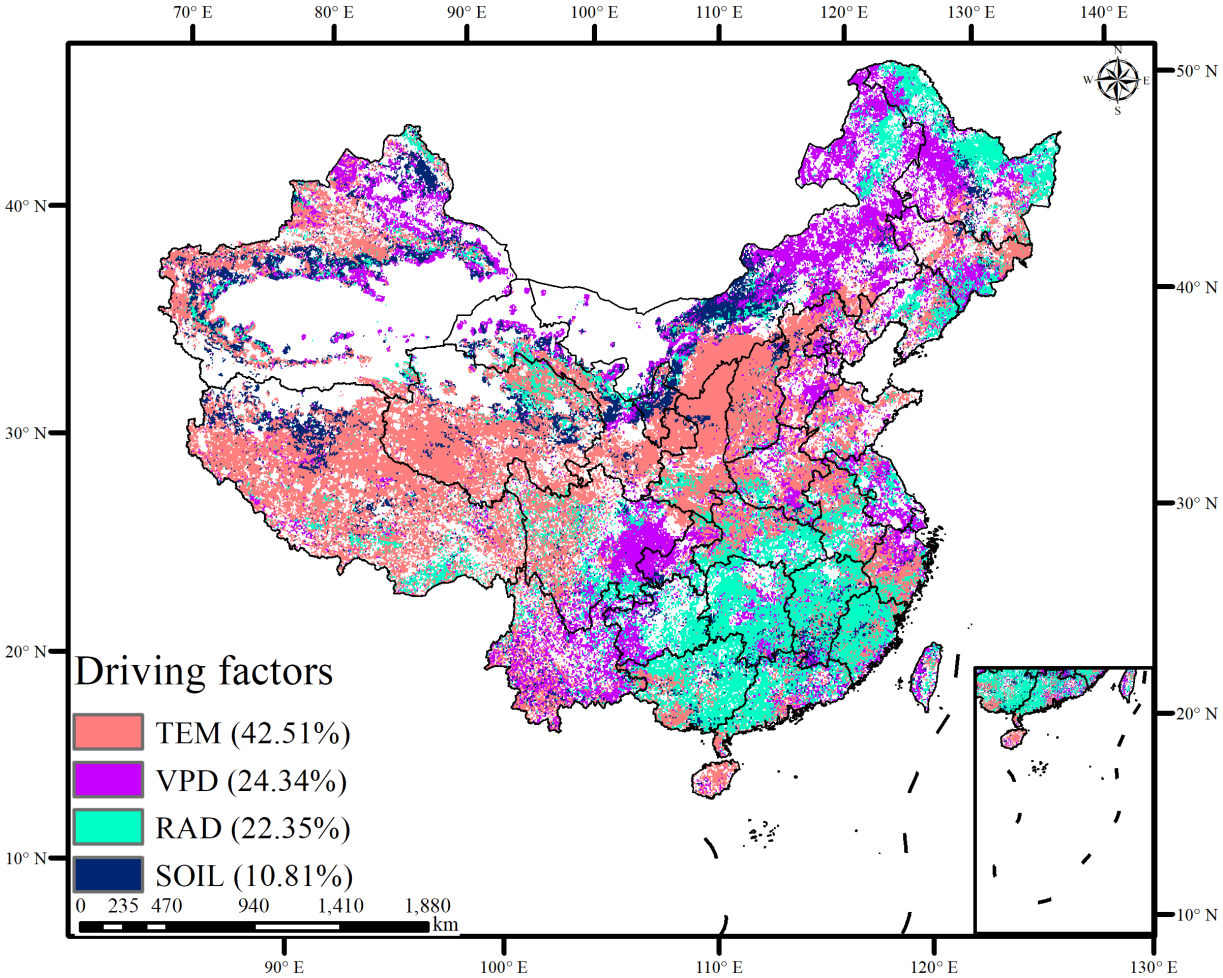
The dominant role of four climate variables to GPP variations (TEM, temperature; VPD, vapor pressure deficit; RAD, radiation; SOIL, soil moisture).

The dominant climatic factors influencing GPP are illustrated in **Figure 7**. Temperature was the primary factor affecting 42.51% of the area, followed by VPD at 24.34%, solar radiation at 22.35%, and soil moisture at 10.81%. Temperature-dominated GPP variations were mainly observed in the Tibetan Plateau and Yellow River Basin, which also exhibited a median stable increase in GPP. VPD-dominated GPP variations were found in the Southwest Karst region, Inner Mongolia, and Heilongjiang, showing a median and non-significant decrease. Soil moisture was the dominant factor in northwestern and southwestern China, leading to a decrease in GPP with a strong stability. Radiation-dominated areas were concentrated in southern China and the northernmost regions, where GPP increases were relatively unstable. Although temperature and radiation promoted GPP variations, temperature contributed significantly to GPP stability.

**Figure 8:**
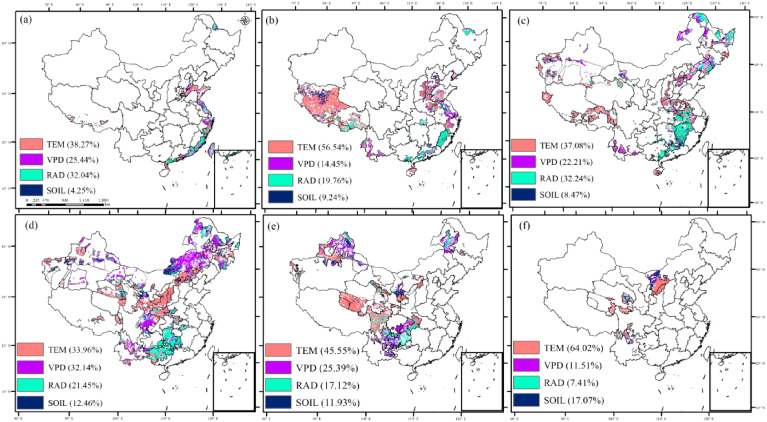
The dominant role of four climate variables to GPP variations in ecological project areas [TEM, temperature; VPD, vapor pressure deficit; RAD, solar radiation; SOIL, soil moisture; **(A)** zero ecological project areas, **(B)** one ecological project area, **(C)** two ecological project areas, **(D)** three ecological project areas, **(E)** four ecological project areas, and **(F)** five ecological project areas].

In regions with overlapping ecological engineering projects, temperature emerged as the primary driver of GPP (**Figure 8**; **Table 3**). Temperature and solar radiation were the main drivers in areas without ecological engineering projects or with fewer overlapping projects, such as Tibet and the Eastern China Coast. VPD and soil moisture contributed to GPP variations in regions with higher overlapping ecological engineering implementations. Specifically, VPD had a higher relative contribution to GPP in the Northeast, Northwest, and Southwest regions, where three or four overlapping ecological projects were prevalent, whereas soil moisture played a more prominent role in regions with five overlapping projects.

**Table 3:** The dominant role of four climate variables to GPP variations in ecological project areas.

Overlapping ecological projects	TEM	VPD	RAD	SOIL
0 ecological project	38.27%	25.44%	32.04%	4.25%
1 ecological project	56.54%	14.45%	19.76%	9.24%
2 ecological projects	37.08%	22.21%	32.24%	8.47%
3 ecological projects	33.96%	32.14%	21.45%	12.46%
4 ecological projects	45.55%	25.39%	17.12%	11.93%
5 ecological projects	64.02%	11.51%	7.41%	17.07%

TEM, temperature; VPD, vapor pressure deficit; RAD, solar radiation; SOIL, soil moisture.

### 3.3 The sensitivity of GPP to climate change

**Figure 9:**
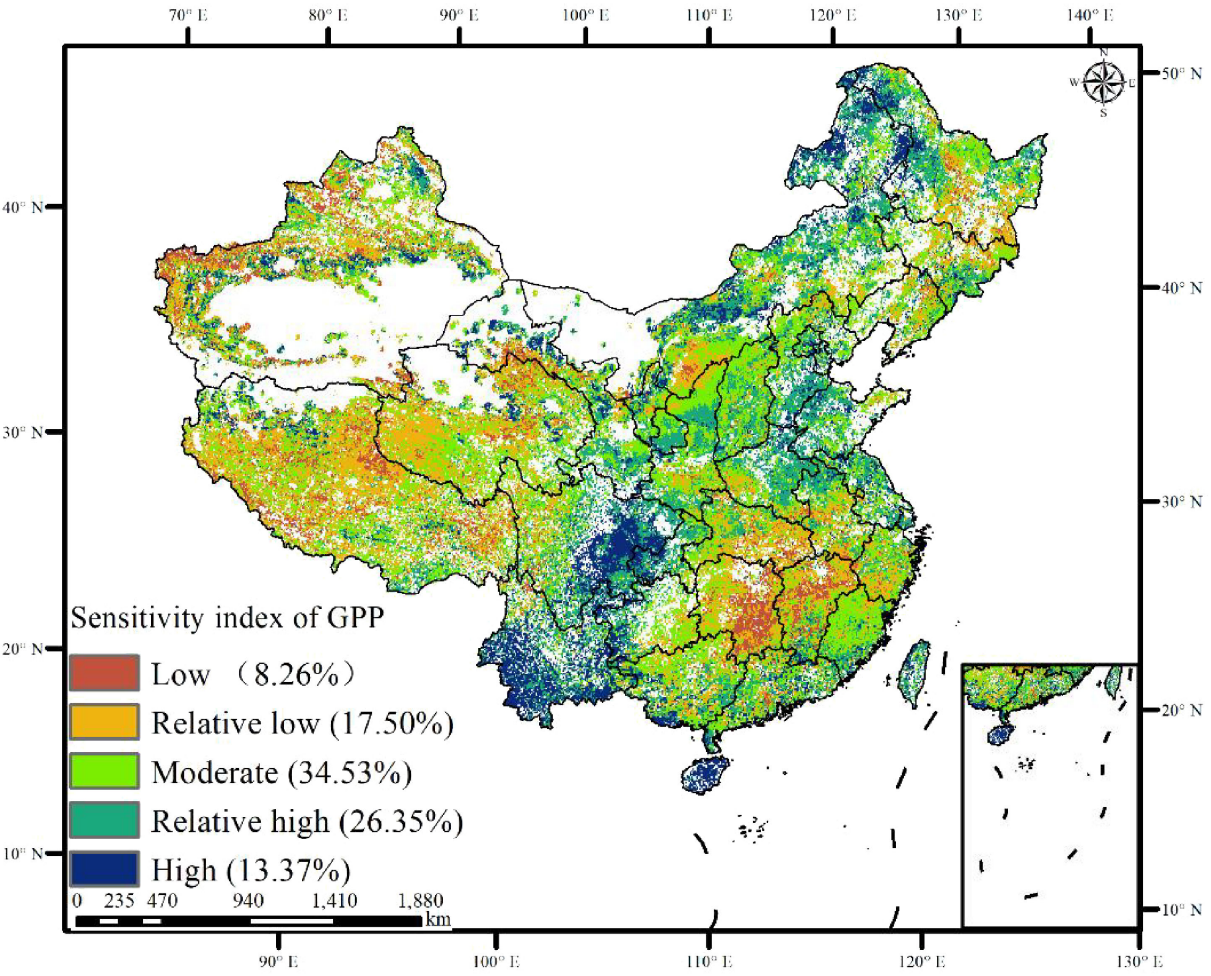
Sensitivity index of GPP in China.

**Figure 10:**
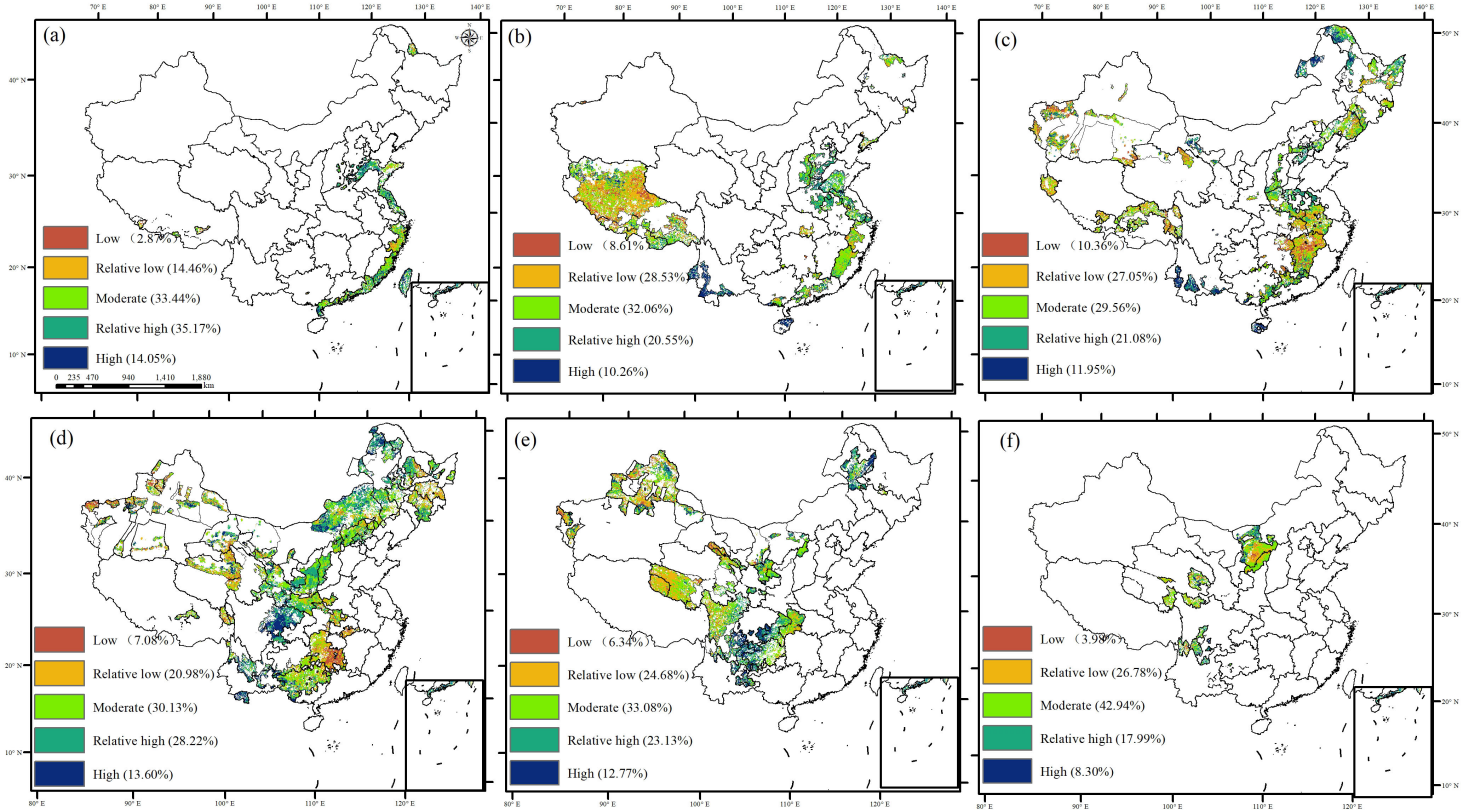
Sensitivity index of GPP in ecological project areas [**(A)** zero ecological project areas, **(B)** one ecological project area, **(C)** two ecological project areas, **(D)** three ecological project areas, **(E)** four ecological project areas, and **(F)** five ecological project areas].

The sensitivity of GPP to climate change was examined using four climate factors [temperature (TEM), vapor pressure deficit (VPD), solar radiation (RAD), and soil moisture (SOIL)] (**Figure 9**). The spatial distribution of the GPP sensitivity index (GSI) corresponded closely with GPP trends and stability. GPP exhibiting a significant increase or stability showed lower sensitivity to climate change, whereas GPP with a decreasing trend or instability exhibited a high sensitivity to climate change. More than 39.27% of China exhibited a high or relatively high GSI, primarily concentrated in the east coast, arid northwest region, Inner Mongolia, and the Southwest Karst region, where no or few ecological projects had been implemented. Conversely, the low and relatively low GSI accounted for 25.76%, mainly observed in the Yangtze River belt, northeastern China, and the Tibetan Plateau, where four or five ecological projects have been implemented (**Figure 10** and **Table 4**). These findings suggest that the spatial distribution of decreasing trends or instability in GPP aligns with highly sensitive areas of GPP.

**Table 4:** Sensitivity index of GPP in ecological project areas.

Overlapping ecological projects	Low	Relatively low	Moderate	Relatively high	High
0 ecological project	2.87%	14.46%	33.44%	35.17%	14.05%
1 ecological project	8.61%	28.53%	32.06%	20.55%	10.26%
2 ecological projects	10.36%	27.05%	29.56%	21.08%	11.95%
3 ecological projects	7.08%	20.98%	30.13%	28.22%	13.60%
4 ecological projects	6.34%	24.68%	33.08%	23.13%	12.77%
5 ecological projects	3.98%	26.78%	42.94%	17.99%	8.30%

## 4 Discussion

### 4.1 Stability of GPP in the overlapping ecological engineering areas

Previous studies have predominantly focused on the GPP dynamics, often overlooking the stability and the impact of overlapping ecological projects on GPP (Seddon et al., 2016; Liu et al., 2023b). Our study revealed that 65.74% of GPP was increasing, primarily in the eastern coastal areas and overlapping implementation of five ecological projects, such as the Grain to Green, the Natural Forest Resource Protection Project, the Three-River Ecological Conservation and Restoration Project, and the Three-North Shelter Forestation Project. Conversely, 20.90% of GPP showed a downward trend, mainly in two specific regions: areas with overlapping implementation of the Three-North Protective Forest Project and the Grain to Green Project, and regions where the Yangtze River Protective Forest Project and the rocky desertification management of the southwestern Karst region overlap. The former areas showed a strong stability, whereas the latter had relatively lower stability. Northwestern China’s arid climate, characterized by water scarcity and high evapotranspiration, is unfavorable for carbon storage (Zhang et al., 2021; Xu et al., 2023). Previous research highlighted that restoration effects have promoted ecosystem recovery in the karst area in southwest China (Yue et al., 2022). However, our findings suggested that the GPP exhibited decreasing but unstable trends in regions where the Yangtze River Protective Forest Project overlaps with the rocky desertification management of the southwestern Karst region. This suggests heightened susceptibility to environmental perturbations. The Southwest Karst region’s troughs and valleys are prone to water leakage and droughts, whereas its depressions and basins experience waterlogging, both of which hinder vegetation growth and reduce carbon sequestration (Wang et al., 2021; Zhu et al., 2023b).

### 4.2 Driving mechanism of GPP in the overlapping ecological engineering areas

The relative response of GPP to environmental perturbations and its driving factors is crucial for ecosystem resilience (Chen et al., 2017; Gao et al., 2018; Piao et al., 2019). Understanding the underlying processes within ecosystems is essential for exploring the sustainability and sensitivity of future carbon cycles and for providing a foundation for nature-based solutions (Zhang et al., 2019; Piao et al., 2022). Previous studies have shown that climate change has direct and significant effects on GPP (Wang et al., 2023; Zhu et al., 2023a). In regions with no or few overlapping ecological projects, temperature and radiation were the primary drivers of GPP changes. However, in regions affected by multiple overlapping ecological projects, water-related factors (VPD and soil moisture) were the primary drivers of GPP changes. This is primarily because the eastern region, with its favorable thermal and hydrological conditions, exhibits high vegetation cover and strong carbon sink capacity, contributing to carbon sink increases even in the absence or minimal presence of ecological projects (Lian et al., 2020). Conversely, in the northwest and southwest of China, harsh climatic conditions and limited moisture are the main factors restricting carbon sink growth; thus, the influence of precipitation-related factors is more pronounced (Liu et al., 2022, 2023a). Therefore, human intervention is necessary to prevent GPP decline and promote its increase in these regions.

### 4.3 The sensitivity of GPP to climate change in overlapping ecological engineering

GPP in areas with overlapping ecological projects was highly sensitive to human activities, particularly in ecological restoration priority areas such as the Loess Plateau and the Yangtze River economic belt. On the Loess Plateau, the implementation of various management measures and ecological construction projects, including slope management, integrated watershed management, ecological restoration programs, and the Grain for Green Program (GGP), has significantly increased forest and grassland vegetation coverage. Since the GGP’s inception, vegetation coverage on the Loess Plateau has risen from 31.6% in 1999 to 67% in 2020, marking a historic transformation from degradation to significant greening (Bai et al., 2019; Chen et al., 2021b). Post-2000, the rate of growth of greenness in the Loess Plateau has outpaced the national average (Kou et al., 2021). This restoration has substantially increased carbon sequestration, transforming the Loess Plateau from a carbon source to a carbon sink, especially in hilly and gully areas where farmland has been returned to forests and grasslands (Zheng et al., 2019). Conversely, the Yangtze River Economic Belt, characterized by dense populations, industrial activities, and high socio-economic development, exhibits a continuous decline in GPP due to extensive construction and arable land use (Jiang et al., 2022).

The decreased and unstable GPP in the China karst and northwest desertification region was highly sensitive to climate change, driven mainly by increased vapor pressure deficit (VPD) and decreased soil moisture. Ecological engineering has been shown to increase vegetation growth and carbon stock in the karst region of southwest China (Tong et al., 2018, 2020). However, this region is still challenged by the distribution of soil and water resources and rapid hydrological changes, resulting in slow soil formation rates, poor water-holding capacity, and low ecological recoverability (Jiao et al., 2024). Low soil water availability and high atmospheric saturation air pressure differences are the main drivers of vegetation greening stress in the karst region (Song et al., 2022). Drought stress due to water scarcity limits the recovery and stability of karst ecosystems, making GPP highly sensitive to climate change (Konings et al., 2017). Additionally, terracing and deforestation on slopes greater than 25° have further damaged the already fragile ecosystems in these areas (Wang et al., 2019, 2021). Vegetation requires significant amounts of energy and nutrients, with soil moisture being crucial, particularly in semi-arid areas (Li et al., 2022). Global climate change is decreasing soil moisture in many regions due to increasing the demand for water evaporation by warming (Carminati and Javaux, 2020). Furthermore, increased vegetation due to climate warming has exacerbated soil drying in arid regions (Deng et al., 2020; Liu et al., 2023c). The decertified area, situated in a climate-sensitive zone with a fragile ecological environment, showed that GPP was highly responsive to soil moisture, with decreased soil moisture being the primary cause of reduced GPP (Song et al., 2022). Soil moisture is essential for the normal growth of vegetation and the sustainable development of agriculture, forestry, and grassland industries in the Loess Plateau region (Li et al., 2022). In semi-arid and arid areas, the relationship between vegetation and soil moisture is more pronounced, and insufficient soil moisture restricts plant growth on the Loess Plateau and in the dry northwest region.

### 4.4 Implications and limitations

This study primarily relies on the reliability of data. While GPP has been accurately estimated in previous studies, recent remote sensing-based global terrestrial carbon, water, and energy budgets remain uncertain (Chen et al., 2021a). Previous integrated flux models have advanced the monitoring of the terrestrial carbon cycle, but they only partially incorporated coupled land-atmosphere fluxes (Yan et al., 2019). Consequently, gaining a comprehensive understanding of the terrestrial carbon cycle alongside other processes remains challenging. These uncoupled scenarios may overlook critical dynamics of fundamental terrestrial biophysical processes, and using different forcing data for individual flux models can lead to internal inconsistencies between carbon and other flux estimates, resulting in significant biases in global annual flux budgets (Zhou et al., 2016). Furthermore, individual flux products can introduce significant biases in estimating ecosystem functional properties (Chang et al., 2024). Therefore, this study utilized an improved satellite-based model of coupled processes, the BESS v2.0. This new version integrated a newly developed ecosystem respiration module and an optimal maximum rate of carboxylation (*V_cmax_*) based model, extending the temporal coverage of the flux dataset from 1982 to 2019. BESS v2.0 products better match flux site observations than other products, ensuring that BESS v2.0 is a reliable and independent set of products from other global satellites and contributing to research related to global carbon, water, and energy budgets in a coupled and comprehensive manner. Notably, carbon flux estimates by BESS in the tropics showed significant differences compared to other models (Zhou et al., 2016). This divergence can be attributed to several factors. First, the reliability of remote-sensed GPP products is compromised by poor-quality forcing data in the tropics due to prolonged cloud contamination. Second, carbon fluxes may not closely follow vegetation indices due to oversaturation but are more sensitive to meteorological factors. Third, the less prominent variation obtained by satellite-derived key forcing data and insufficient spatial coverage of flux site observations in tropical forests likely contributed to the comparatively low performance in some data-driven models (Jiang and Ryu, 2016). Additionally, the significant bias over croplands may be partly due to the lack of distinction between C3 and C4 crops in some machine-learning GPP products (Jiang and Ryu, 2016). We also noted that other proxies, such as solar-induced fluorescence (SIF), may perform well as suggested. However, the available data from SIF start only from 2000, limiting our understanding of long-term GPP dynamics (Jiang and Ryu, 2016). Future research should consider combining SIF with vegetation indices to extend the SIF-GPP relationship back to the start of the remote sensing era in the 1980s.

Investigating the impact of climatic variations on interannual variability of land carbon flux remains crucial (Piao et al., 2020). However, quantifying the influence of other variables such as land cover changes (urbanization) and vegetation growth stages on GPP trends proves challenging due to their complexity (Seddon et al., 2016). This study focused exclusively on climate change effects; therefore, we did not present results on land cover changes or vegetation growth stages directly driving GPP variations. Nevertheless, these stages may modulate vegetation responses to climatic shifts. Notably, recent observations suggest a weakening relationship between GPP and temperature (Piao et al., 2014). Hence, future research should adopt novel methodologies to comprehensively assess GPP changes. While our investigation primarily addressed annual-scale climatic controls, ongoing discussions persist regarding seasonal variability. Environmental impacts during spring, summer, and autumn may differ significantly. Thus, future studies could explore how GPP responds to climate fluctuations across diverse temporal scales.

Despite some inevitable limitations, we point out that our results can help future researchers better understand the stability of GPP and its driving mechanisms, providing scientific support for ecological protection projects.

## 5 Conclusions

This study investigated the stability and sensitivity of GPP to climate variability in China in the last four decades and then explored the driving mechanisms of GPP variations. The meaningful findings are as follows: GPP generally showed an increased trend, with less stability in Eastern regions, whereas a decreased trend with higher stability was observed in Western and Southwest China. Notably, GPP stability was highest (74.58%) in areas with five overlapping ecological projects: Grain to Green, Natural Forest Resource Protection, Three-River Ecological Conservation and Restoration, Return Grazing to Grassland, and Three-North Shelter Forestation. In regions with minimal or no overlapping ecological projects, temperature and radiation mainly influenced GPP variations. In contrast, water-related factors (VPD and soil moisture) significantly affected GPP in areas with multiple overlapping ecological projects. Particularly in areas impacted by the simultaneous implementation of the five projects, GPP showed an increasing trend (81.97%) and lower sensitivity (3.98%) to climate change.

## Data Availability

The original contributions presented in the study are included in the article/**Supplementary Material**. Further inquiries can be directed to the corresponding author.

## Supplementary material

Supplementary Figure S1 The trend of driving factors on GPP from 1982 to 2019 in China [(A) Temperature; (B) VPD; (C) radiation; (D) soil moisture].
