# Supplementary figures and images for: Stability of gross primary productivity and its sensitivity to climate variability in China
Source: Front Plant Sci. 2024 Sep 6;15:1440993. doi: 10.3389/fpls.2024.1440993 (PMC11412862; doi:10.3389/fpls.2024.1440993)

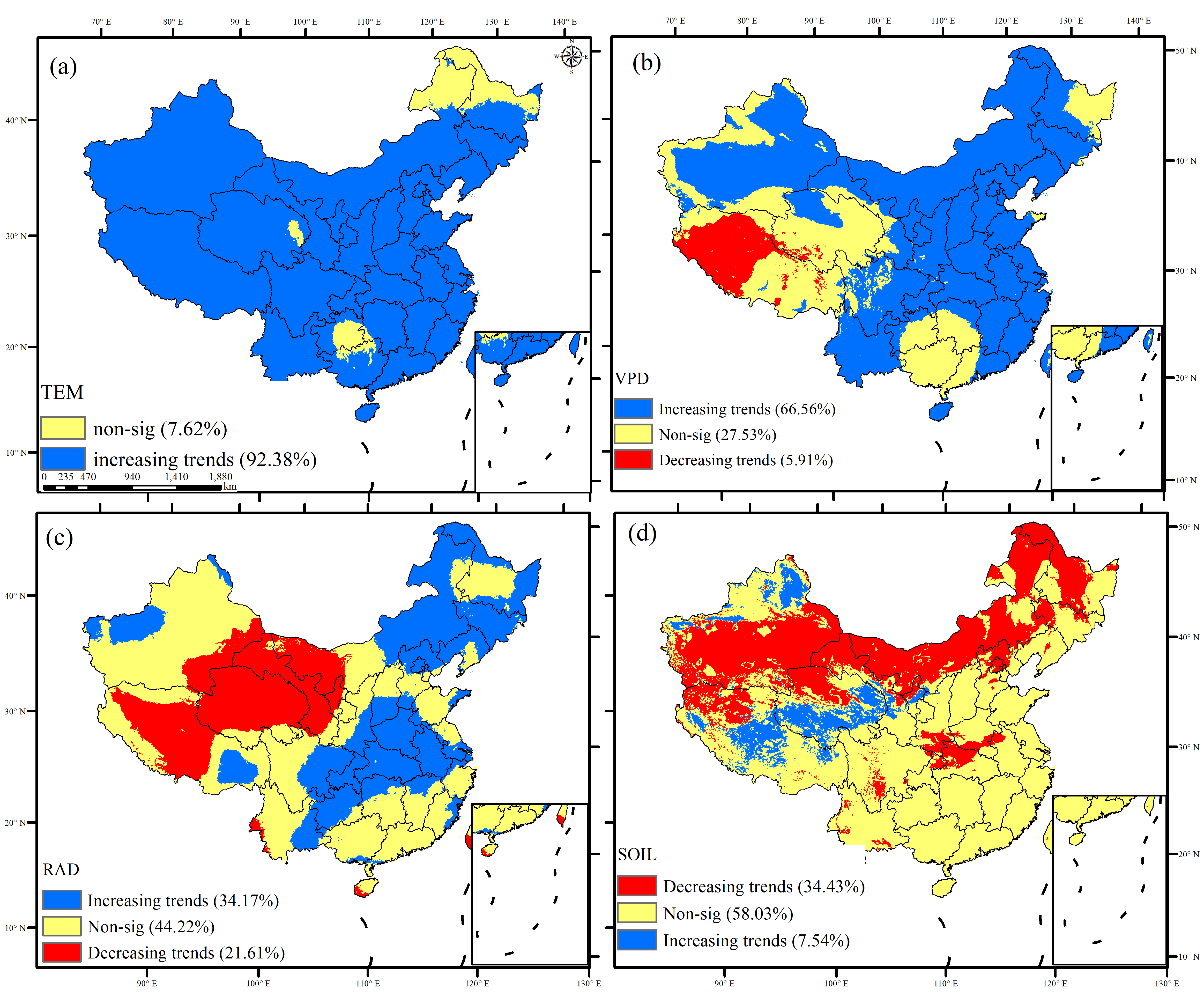

Supplement: Supplementary Figure S1 — The trend of driving factors on GPP from 1982 to 2019 in China [(A) Temperature; (B) VPD; (C) radiation; (D) soil moisture]. [file Image1.tif]
